# Non-canonical protein-DNA interactions identified by ChIP are not artifacts

**DOI:** 10.1186/1471-2164-14-254

**Published:** 2013-04-15

**Authors:** Richard P Bonocora, Devon M Fitzgerald, Anne M Stringer, Joseph T Wade

**Affiliations:** 1 Wadsworth Center, New York State Department of Health, Albany, NY, 12208, USA; 2 Department of Biomedical Sciences, University at Albany, Albany, NY, 12201, USA

**Keywords:** ChIP-chip, ChIP-seq, σ^32^

## Abstract

**Background:**

ChIP-chip and ChIP-seq are widely used methods to map protein-DNA interactions on a genomic scale *in vivo*. Waldminghaus and Skarstad recently reported, in this journal, a modified method for ChIP-chip. Based on a comparison of our previously-published ChIP-chip data for *Escherichia coli* σ^32^ with their own data, Waldminghaus and Skarstad concluded that many of the σ^32^ targets identified in our earlier work are false positives. In particular, we identified many non-canonical σ^32^ targets that are located inside genes or are associated with genes that show no detectable regulation by σ^32^. Waldminghaus and Skarstad propose that such non-canonical sites are artifacts, identified due to flaws in the standard ChIP methodology. Waldminghaus and Skarstad suggest specific changes to the standard ChIP procedure that reportedly eliminate the claimed artifacts.

**Results:**

We reanalyzed our published ChIP-chip datasets for σ^32^ and the datasets generated by Waldminghaus and Skarstad to assess data quality and reproducibility. We also performed targeted ChIP/qPCR for σ^32^ and an unrelated transcription factor, AraC, using the standard ChIP method and the modified ChIP method proposed by Waldminghaus and Skarstad. Furthermore, we determined the association of core RNA polymerase with disputed σ^32^ promoters, with and without overexpression of σ^32^. We show that (i) our published σ^32^ ChIP-chip datasets have a consistently higher dynamic range than those of Waldminghaus and Skarstad, (ii) our published σ^32^ ChIP-chip datasets are highly reproducible, whereas those of Waldminghaus and Skarstad are not, (iii) non-canonical σ^32^ target regions are enriched in a σ^32^ ChIP in a heat shock-dependent manner, regardless of the ChIP method used, (iv) association of core RNA polymerase with some disputed σ^32^ target genes is induced by overexpression of σ^32^, (v) σ^32^ targets disputed by Waldminghaus and Skarstad are predominantly those that are most weakly bound, and (vi) the modifications to the ChIP method proposed by Waldminghaus and Skarstad reduce enrichment of all protein-bound genomic regions.

**Conclusions:**

The modifications to the ChIP-chip method suggested by Waldminghaus and Skarstad reduce rather than increase the quality of ChIP data. Hence, the non-canonical σ^32^ targets identified in our previous study are likely to be genuine. We propose that the failure of Waldminghaus and Skarstad to identify many of these σ^32^ targets is due predominantly to the lower data quality in their study. We conclude that surprising ChIP-chip results are not artifacts to be ignored, but rather indications that our understanding of DNA-binding proteins is incomplete.

## Background

ChIP-chip (sometimes referred to as ChIP-on-chip) and ChIP-seq are widely-used genomic methods that combine chromatin immunoprecipitation (ChIP) with microarrays and deep sequencing, respectively, to map protein-DNA interactions *in vivo*[1]. The genome-wide binding profiles of hundreds of proteins have been mapped using ChIP-chip and ChIP-seq in organisms ranging from bacteria to humans. ChIP-chip/ChIP-seq often identifies non-canonical target regions for DNA-associated proteins, i.e. target regions that are inconsistent with our current understanding of the protein being studied. In many cases, these discoveries have provided new insight into the function of those proteins. In bacteria, many transcription factor (TF) binding sites identified using ChIP-chip/ChIP-seq are located in “unexpected” genomic regions: (i) upstream of genes whose described function is seemingly unconnected to the described function of the TF [2-4], (ii) upstream of genes whose expression does not change detectably when the TF-encoding gene is mutated [2,4-8], (iii) inside genes [2-4,9-13], and (iv) far from any DNA sequences that are close matches to the known consensus binding site [2,3,8,14,15]. In most cases, the significance of these observations is unclear, although they suggest that (i) gene annotations are often incomplete, (ii) TFs often function redundantly, such that expression of the regulated gene does not change unless multiple TF-encoding genes are deleted, (iii) TFs often regulate the expression of non-coding RNAs that initiate within genes [16], and (iv) TFs often bind DNA cooperatively such that the DNA sequence requirements are altered or relaxed.

Our published ChIP-chip study of σ^32^, an alternative σ factor in *E. coli*, led to the identification of 22 putative σ^32^ binding sites within genes [11]. These represent ~25% of all the σ^32^ binding sites we identified. All but 2 of the gene-internal promoters are >300 bp from an annotated translation start codon. We proposed that RNA polymerase (RNAP) associated with σ^32^ (RNAP:σ^32^) often binds to promoter elements within genes and initiates transcription of non-coding RNAs in either the sense or antisense orientation. We confirmed this for three examples that we examined in more detail. Furthermore, five of the σ^32^ binding sites within genes are immediately adjacent to genes identified in previous studies as being upregulated by σ^32^, but for which no promoter could be identified in the upstream region [17,18]. Our ChIP-chip data also permitted identification of 65 σ^32^ binding sites in intergenic regions, 26 of which are not associated with genes identified in either of two transcriptomic studies of σ^32^[17,18]. Thus, many of the sites of σ^32^ association we identified are non-canonical.

In a recent study published in this journal, Waldminghaus and Skarstad describe modifications to the standard ChIP-chip procedure [19]. The key modifications are avoiding the use of Spin-X filter columns during immunoprecipitation (IP) wash steps, including an RNase treatment following the IP, and collecting reference material after the IP rather the traditional “input” starting chromatin. Waldminghaus and Skarstad propose that the standard ChIP-chip method results in identification of false positives that are eliminated when using the modified method. Waldminghaus and Skarstad demonstrated their modified ChIP-chip procedure by performing ChIP-chip of *E. coli* σ^32^. They identified many fewer target regions for σ^32^ than our earlier study. We will refer to the 46 σ^32^ target regions identified in our previous study but not by Waldminghaus and Skarstad as “Disputed σ^32^ targets” (DSTs). DSTs are enriched for non-canonical σ^32^ binding sites. Specifically, 16 of the 46 DSTs are located inside genes or between convergently transcribed genes, and 21 DSTs are located in intergenic regions but are not associated with genes identified in transcriptomic studies of σ^32^[17,18]. We have reanalyzed our published ChIP-chip datasets and those of Waldminghaus and Skarstad. This reanalysis demonstrates low reproducibility in the datasets of Waldminghaus and Skarstad. We also used targeted ChIP/qPCR to directly compare the standard and modified ChIP methods. We demonstrate that non-canonical targets of σ^32^ are real and that the lower data quality and deficiencies in the modified ChIP method are sufficient to explain the absence of DSTs in the list of σ^32^ targets generated by Waldminghaus and Skarstad.

## Results and discussion

### Existing evidence that DSTs are genuine sites of σ^32^ association

Waldminghaus and Skarstad suggest that DSTs are artifacts that result from non-specific IP of RNA that is then amplified by Klenow DNA polymerase during sample preparation for ChIP-chip [19]. However, there are several features of DSTs that are consistent with them being genuine sites of σ^32^ association and inconsistent with them being artifacts resulting from amplification from RNA:

(i) Nine of the DSTs (*mfd*, *phoP*, *ldhA*, *recF*, *narP*, *holC*, *glnS*, *ileS*, and *yfjN*) are σ^32^ targets identified in independent studies that did not involve ChIP [17,18]. With the exception of the DSTs inside *yfjN* and *recF*, these would all be considered canonical σ^32^ binding sites, i.e. located in an intergenic region upstream of a gene known from previous studies to be transcribed by σ^32^[17,18].

(ii) Our previous study included validation of three non-canonical DSTs (between *tdk* and *ychG*, within *dhaM*, and within *ydeP*) using ChIP/qPCR [11]. This method does not involve amplification of ChIP DNA using Klenow DNA polymerase. Furthermore, we demonstrated heat shock-dependent increases of σ^32^ association with all three regions [11].

(iii) Although many DSTs are located inside genes, there are significantly more DSTs located in intergenic regions than expected by chance (Binomial Test *p* = 0.00033).

Note that, for all the analyses described herein, we have excluded the two DSTs that are located in repetitive sequence (*yibA* and *yrdA*; see Conclusions).

### Comparison of data quality between our data and those of Waldminghaus and Skarstad

**Figure 1:**
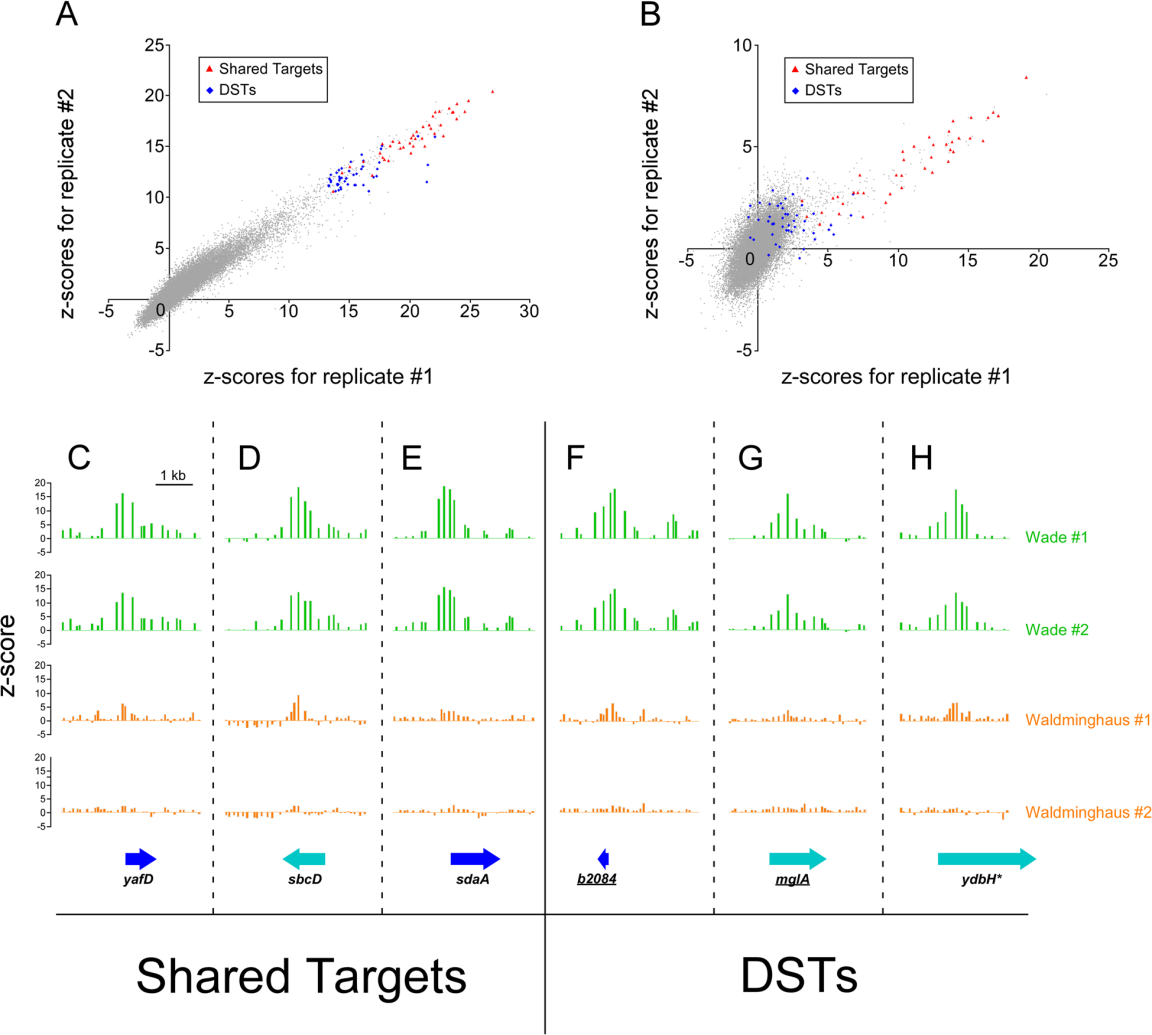
**Normalized ChIP-chip data. (A+B).** Plots of z-scores (see methods) for duplicate ChIP-chip datasets from (**A**) our study and (**B**) that of Waldminghaus and Skarstad. Each graph shows z-scores for one replicate dataset plotted against z-scores for the other replicate dataset. Each datapoint shown represents one microarray probe. Datapoints in red represent probes that correspond to σ^32^ binding sites identified in both studies. Datapoints in blue represent probes that correspond to DSTs. For the Waldminghaus and Skarstad datasets, the nearest probe to the DST coordinate was chosen (the microarray probes for each study are different). Note that some high-scoring probes were not identified as σ^32^ binding sites because they are adjacent to a probe with an even higher score. (**C-H**). Plots indicating z-scores for probes within specific genomic regions. (**C-E**). Regions containing sites of σ^32^ association identified by our previous work and by Waldminghaus and Skarstad. (**F-H**). Regions containing DSTs. Data for two replicate experiments from the work of Waldminghaus and Skarstad are shown in orange. Data for two replicate experiments from our previous study are shown in green. Values plotted are the z-scores (see Methods) for each microarray probe in the region. For each plot, associated genes are indicated as blue arrows. Light blue arrows indicate genes for which the site of σ^32^ association is intragenic, i.e. non-canonical σ^32^ promoters. Genes with underlined names were not detected in transcriptomic studies of σ^32^[17,18]. The asterisk indicates a ChIP-chip peak that was assigned to the intergenic region upstream of *ldhA*, the adjacent gene [11]. Note that *ldhA* was identified as being upregulated by overexpression of σ^32^[17].

The disparity between the σ^32^ targets identified in the two studies led us to compare the quality of the ChIP-chip data. For each dataset we used an established method to estimate the null distribution of ChIP-chip signals [20,21]. Specifically, we determined the modal value and used the probes with scores at or below this value to fit a normal distribution. Using this fitted normal distribution we determined the mean and standard deviation of the null distribution. This allowed us to calculate z-scores (number of standard deviations from the mean) for each microarray probe, thus providing a measure of dynamic range that is independent of the absolute ChIP-chip signals, which have arbitrary units. Scatter plots of z-scores for the duplicate datasets from each study are shown in Figure 1A-B. These scatter plots demonstrate several key features of the datasets from each study:

(i) The two replicate datasets for our study correlate very well (Spearman Correlation Coefficient of 0.93) whereas those of Waldminghaus and Skarstad correlate less well (Spearman Correlation Coefficient of 0.64).

(ii) One of the datasets of Waldminghaus and Skarstad has a substantially lower dynamic range than the other. Several of the targets identified in both studies have z-scores within the noise for this replicate.

(iii) Although the dynamic range of one Waldminghaus and Skarstad dataset is high, the vast majority (~98.5%) of the probes have z-scores lower than 3, suggesting that these datasets are effective at identifying strong protein-DNA interactions but not weaker interactions.

(iv) Although they were not called as targets, DSTs have significantly higher z-scores for the datasets of Waldminghaus and Skarstad than expected by chance (Mann Whitney U Test *p* < 1e^-30^ for each replicate dataset).

We conclude that our ChIP-chip data are of substantially higher quality with respect to both dynamic range and reproducibility. Figure 1C-H shows normalized ChIP-chip data for replicate datasets from both studies for six selected genomic regions. These data further demonstrate the differences in reproducibility and dynamic range between the two studies. The genomic regions shown include DSTs and non-canonical targets (inside genes and/or no detectable regulation in transcriptomic studies).

Several factors likely contribute to the difference in data quality between the two studies. First, we used a TAP-tagged derivative of σ^32^ whereas Waldminghaus and Skarstad used an antibody raised against the native protein. Second, our heat shock conditions (50°C for 10 minutes) were different to those of Waldminghaus and Skarstad (43°C for 5 minutes). Third, as described below, the modifications to the ChIP method reduce the sensitivity of the assay.

### ChIP/qPCR validation of DSTs

**Figure 2:**
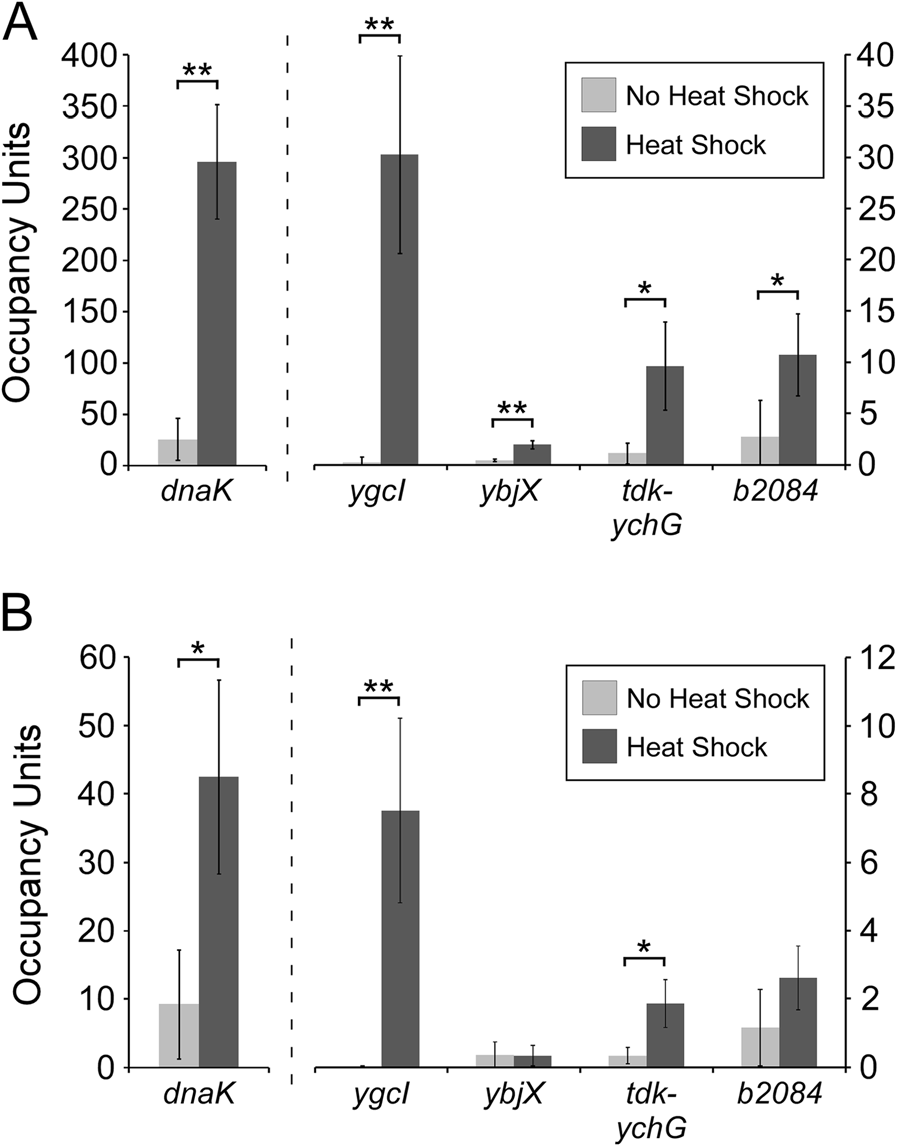
**ChIP/qPCR of σ**^**32 **^**using the standard and modified ChIP methods.** ChIP/qPCR measurement of σ^32^ association with *dnaK* and four DSTs for cells grown without heat shock (light gray bars) or with heat shock (dark gray bars). Data are shown using (**A**) the standard ChIP method, and (**B**) the modified ChIP method described by Waldminghaus and Skarstad [19]. Occupancy units represent background-subtracted enrichment of target regions relative to a control region. Error bars represent the standard deviation from three independent biological replicates. Significant differences between no heat shock and heat shock values are indicated (*p < 0.05, **p < 0.01; one-tailed t-test). Note that the left y-axis is specific for *dnaK* since these occupancy scores are considerably higher than those for the other regions tested.

We used ChIP/qPCR with the standard and modified ChIP methods to measure association of σ^32^ with four DSTs in cells before and after heat shock. As a positive control, we measured association of σ^32^ with the region upstream of *dnaK*, a well-established σ^32^ target [17,18] identified both in our study and that of Waldminghaus and Skarstad. We used cells expressing an N-terminally FLAG-tagged copy of σ^32^ expressed from its native locus (our earlier study used a C-terminally TAP-tagged copy of σ^32^). Using the standard ChIP method, we observed significant association of σ^32^ with all regions tested and a significant increase in σ^32^ association with all regions tested following heat shock (Figure 2A). Previous ChIP-seq studies have revealed biases in the level of some genomic regions in input DNA, the most common control sample for ChIP experiments [22-24]. In the case of ChIP-chip, this bias is likely to be due to nucleosomes, and is hence specific to eukaryotes [23,24]. Nevertheless, we wished to rule out the possibility that DSTs were identified as a result of input biases. Therefore, we repeated the ChIP/qPCR using an untagged strain. We observed no significant ChIP/qPCR signal for any region tested (Additional file 1: Supplementary Data). We conclude that all four DSTs tested are genuine sites of σ^32^ binding.

We compared the standard ChIP method with the modified method proposed by Waldminghaus and Skarstad. Importantly, ChIP with the modified method used the same sonicated, cross-linked cell extracts as the standard method. Using the modified method, we detected significant σ^32^ association with the region upstream of *dnaK* (Figure 2B), and association increased significantly following heat shock (Figure 2B). However, the absolute ChIP signal was substantially lower than that observed using the standard ChIP method (Figure 2A). Thus, the modified ChIP method has a decreased sensitivity relative to the standard method. Using the modified ChIP method we detected significant association of σ^32^ following heat shock with three of the four DSTs tested (Figure 2B). We also observed a significant reduction in σ^32^ association in the absence of heat shock at two of these DSTs (Figure 2B). Thus, even with the decreased sensitivity of the modified ChIP method, three of the four DSTs tested were validated as genuine sites of σ^32^ association. We believe that we were unable to detect significant association of σ^32^ with the fourth DST, *ybjX*, due to the substantial decrease in sensitivity relative to the standard ChIP method. We note that the ChIP signal for *ybjX* was the lowest of all the regions tested using the standard method (Figure 2A). We conclude that the reduced sensitivity of the modified ChIP method prevented Waldminghaus and Skarstad from identifying DSTs as sites of σ^32^ association. This is consistent with the observation that DSTs have above average ChIP-chip scores in the Waldminghaus and Skarstad datasets (Figure 1B).

**Figure 3:**
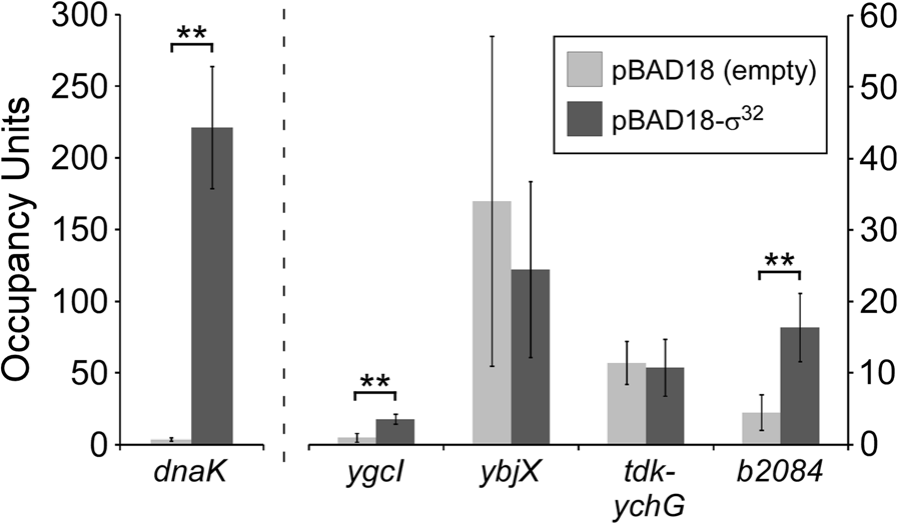
**ChIP/qPCR of core RNAP (β) for cells grown with or without σ**^**32 **^**overexpression.** ChIP/qPCR measurement (standard method) of σ^32^ association with *dnaK* and four DSTs for cells grown without σ^32^ overexpression (light gray bars) or with σ^32^ overexpression (dark gray bars). Occupancy units represent background-subtracted enrichment of target regions relative to a control region. Error bars represent the standard deviation from three independent biological replicates. Significant differences between values for no σ^32^ overexpression and values for σ^32^ overexpression are indicated (*p < 0.05, **p < 0.01; one-tailed t-test). Note that the left y-axis is specific for *dnaK* since these occupancy scores are considerably higher than those for the other regions tested.

As an independent assessment of σ^32^ association with DSTs, we measured association of core RNAP (β subunit) with *dnaK* and the four DSTs described above, with and without overexpression of σ^32^ from a plasmid. Association of β with *dnaK* and two DSTs was significantly higher in cells overexpressing σ^32^ as compared to those with empty vector (Figure 3). This provides independent validation of the association of σ^32^ with these regions. Two of the DSTs tested showed no significant difference in the association of β between cells overexpressing σ^32^ and those with empty vector. In the case of *ybjX*, we propose that the lack of increase in RNAP levels is due to the relatively low association of σ^32^ (Figure 2A). Thus, association of RNAP:σ^32^ may not significantly increase the overall association of RNAP in the presence of a relatively high level of RNAP that is independent of σ^32^ (presumably RNAP:σ^70^). Consistent with our ChIP/qPCR data, *ybjX* expression was not detectably increased by σ^32^ overexpression in two transcriptomic studies [17,18]. In the case of *tdk*/*ychG*, we propose that RNAP:σ^32^ binds this region specifically during heat shock but not following σ^32^ over-expression without heat shock, perhaps due to the requirement for other heat shock-induced/activated proteins.

### ChIP method comparison for AraC

**Figure 4:**
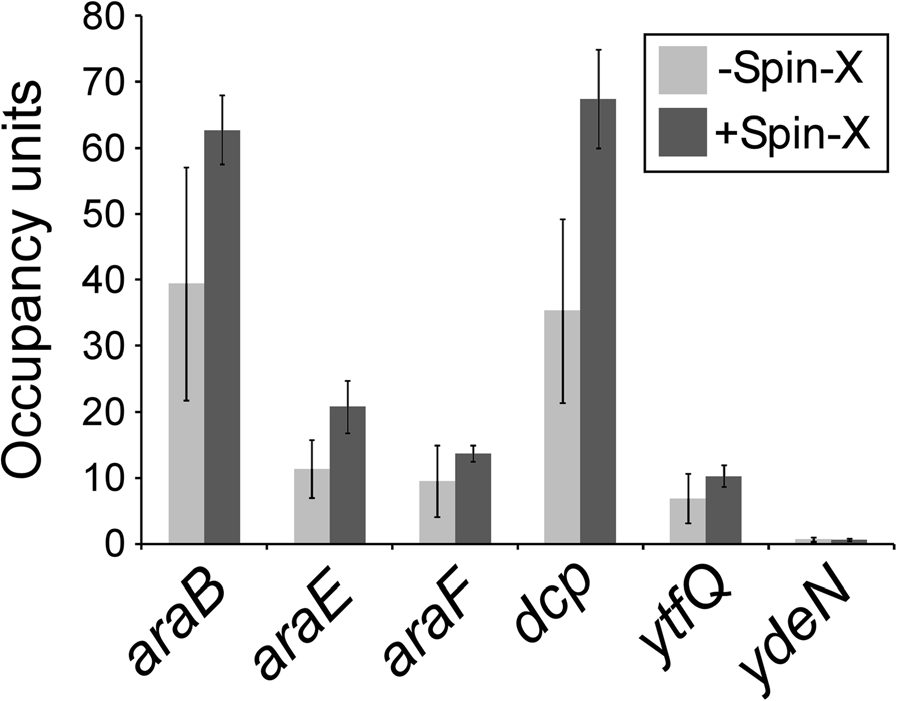
**ChIP/qPCR of AraC using the standard method with or without Spin-X columns.** ChIP/qPCR measurement of AraC association with six known AraC target regions for cells grown in the absence of arabinose. Data are shown for ChIP performed without Spin-X columns (light gray bars) or with Spin-X columns (dark gray bars). Note that the region upstream of *ydeN* is only bound by AraC in the presence of arabinose and hence serves as a control in this experiment. Occupancy units represent background-subtracted enrichment of target regions relative to a control region. Error bars represent the standard deviation from three independent biological replicates. Note that data for ChIP/qPCR using Spin-X columns (dark gray bars) will be described elsewhere (Stringer, A.M., Currenti, S.A., Bonocora, R.P., Baranowski, C., Petrone, B.L., Singh, N., Palumbo, M.J., Reilly, A.E., Zhang, Z., Erill, I. and Wade, J.T.: Comprehensive genomic analysis of the *Escherichia coli* and *Salmonella enterica* AraC regulons; in preparation) and serve only as a reference for data from ChIP/qPCR without Spin-X columns.

The comparison of the ChIP methodologies described above demonstrates that the modified ChIP method is less sensitive. There are multiple changes to the standard method, so it is unclear which specific change(s) results in the decreased sensitivity. One significant change in the method described by Waldminghaus and Skarstad is the omission of Spin-X columns during the IP wash steps. We directly assessed the importance of Spin-X columns by measuring association of AraC (C-terminally FLAG-tagged) with target regions in *E. coli* using ChIP/qPCR performed either with or without Spin-X columns. The use of Spin-X columns increased the ChIP/qPCR signal for all regions tested but qualitatively the data are the same for both methods (Figure 4). Importantly, we detected association of AraC with a non-canonical target within the *dcp* gene using both methods (Figure 4). This site of AraC association is hundreds of base pairs from either end of the gene and there is no detectable change in transcription of *dcp* or association of RNAP at this region following deletion of *araC* and/or addition of arabinose (Stringer, A.M., Currenti, S.A., Bonocora, R.P., Baranowski, C., Petrone, B.L., Singh, N., Palumbo, M.J., Reilly, A.E., Zhang, Z., Erill, I. and Wade, J.T.: Comprehensive genomic analysis of the Escherichia coli and Salmonella enterica AraC regulons; in preparation). Thus, the Spin-X column-free ChIP method detects association with non-canonical target regions, although association with all target regions is reduced relative to the standard ChIP method. In a control experiment using an untagged strain, we observed no significant ChIP/qPCR signal (using the standard ChIP method) for any region tested (Additional file 1: Supplementary Data).

## Conclusions

We conclude that Waldminghaus and Skarstad failed to identify DSTs not because of an improvement in the ChIP methodology, but because of lower data quality. Consistent with this, the majority of DSTs showed relatively low association of σ^32^ in our study: when ranked by the level of σ^32^ association, 36 of the bottom 43 targets are DSTs (Figure 1A) [11]. Furthermore, DSTs have significantly higher signal in the Waldminghaus and Skarstad datasets than expected by chance (*p* < 1e^-30^; Figure 1B), consistent with the idea that these regions represent true binding sites for σ^32^ but fall below the detection threshold of this analysis. We note that Waldminghaus and Skarstad did not present any σ^32^ ChIP data generated using the standard methodology, precluding direct comparison of our work, nor did they use ChIP/qPCR with their modified method to measure association of σ^32^ with specific target regions [19]. Furthermore, Waldminghaus and Skarstad demonstrated a dramatic improvement in ChIP-chip data for SeqA using the modified ChIP method [19], but their data is very similar to that generated using the standard ChIP method by another group [25].

Our comparison of ChIP-chip datasets highlights the importance of data quality for correct identification of protein-DNA interactions. Guidelines for ChIP-chip and ChIP-seq experimental and analytical approaches have been described previously [26,27]. Key components of these methods that are especially relevant to our own study are the comparison of replicates, the choice of control, and the importance of repetitive sequence. Current guidelines for ChIP-seq recommend the use of only two independent biological replicates [27], but also stress the importance of reproducibility. As shown in Figure 1B, the poor reproducibility of the Waldminghaus and Skarstad datasets is likely to be a major cause of their failure to identify DSTs as regions truly bound by σ^32^. Recommended controls are either input DNA or ChIP-enriched DNA from an untagged strain (when using an epitope-tagged protein). Waldminghaus and Skarstad instead used DNA left in the supernatant after the initial IP, acknowledging that this DNA would be de-enriched for target regions. While this may increase the apparent signal, we caution against this approach as the ChIP-chip or ChIP-seq signals may not accurately reflect the actual level of binding. Finally, Waldminghaus and Skarstad highlighted the importance of treating repetitive DNA sequences with caution when interpreting ChIP-chip (or ChIP-seq) datasets. In the case of σ^32^, two of the ChIP peaks identified in our earlier study overlap repetitive regions. It is impossible to determine from ChIP-chip data alone whether σ^32^ associates with one or all of the repetitive regions. Since this caveat applies to repetitive sequences in any ChIP-chip or ChIP-seq experiment, we echo the sentiment expressed by Waldminghaus and Skarstad and caution against analysis of sequences in these regions.

Many ChIP-chip studies have revealed the existence of unexpected protein-DNA interactions. For example, ChIP-chip studies in bacteria have demonstrated that transcription factors often bind to sites within genes, sites without a recognizable motif, and sites that are not associated with described regulation by the transcription factor [15]. This is one of the great strengths of ChIP-chip and ChIP-seq, since these non-canonical binding sites often cannot be identified using other genomic approaches such as transcription profiling. In the case of σ^32^, our data provide strong evidence that RNAP:σ^32^ initiates transcription of many RNAs from within genes, and our original study described three such examples in greater detail [11]. The function of intragenic transcripts in bacteria is poorly understood, although several antisense transcripts have been shown previously to regulate expression of the overlapping mRNA [28]. Our own studies have revealed pervasive antisense transcription in *E. coli*[16], and this has since been observed in several other bacterial species [28]. Intriguingly, many ChIP-chip studies of bacterial DNA-binding TFs have revealed sites of association inside genes [10,15], suggesting regulation of intragenic transcripts. Similar phenomena have been observed in eukaryotes, including human cells [29,30]. Other types of non-canonical transcription factor binding sites, i.e. sites without a recognizable motif and sites that are not associated with described regulation by the transcription factor, are also poorly understood. However, sites without a recognizable motif could be explained by indirect association with DNA (detectable using ChIP) [15,31] or cooperative interactions with other DNA-binding proteins [32]. Sites that are not associated with described regulation by the transcription factor could be explained by combinatorial regulation by multiple, redundant transcription factors. In the case of σ^32^, our data suggest that many σ^32^ promoters are not associated with detectable regulation using transcriptomic approaches due to a high basal level of transcription, or a specific requirement for heat shock conditions.

It is important to note that Waldminghaus and Skarstad identified many non-canonical σ^32^-target regions in their study. Specifically, Waldminghaus and Skarstad detected σ^32^ association upstream of four genes whose expression was not detectably upregulated by overexpression of σ^32^ in either of two transcriptomic studies (*yafU*, *rpsL*, *yjhI*, and *fimB*) [17,18], and six sites of σ^32^ association within genes or between convergently transcribed genes (*yfbM/yfbN*, *yfjU*, *ypjA*, *sbcD*, *cycA*, and *macB*) [19]. Waldminghaus and Skarstad suggest that “surprising”, non-canonical protein-DNA interactions are often artifacts. We caution against this dogmatic approach. Artifacts can arise from ChIP-chip and ChIP-seq experiments; however, with the appropriate experimental and analytical methods, and with the appropriate controls, it is possible to identify protein-DNA interactions with high confidence. Atypical binding sites identified using these methods may indicate novel functions for well-studied proteins. These binding sites should not be dismissed, but rather should be the focus of additional studies.

## Methods

### Strains and plasmids

*E. coli* MG1655 *rpoH*-NFLAG containing the *rpoH* gene at its native chromosomal location fused to three FLAG tags was constructed using FRUIT [33]. Primer sequences are available on request. Construction of MG1655 with C-terminally FLAG-tagged AraC (AMD187) will be described elsewhere (Stringer, A.M., Currenti, S.A., Bonocora, R.P., Baranowski, C., Petrone, B.L., Singh, N., Palumbo, M.J., Reilly, A.E., Zhang, Z., Erill, I. and Wade, J.T.: Comprehensive genomic analysis of the *Escherichia coli* and *Salmonella enterica* AraC regulons; in preparation).

pRB1 for expression of the *rpoH* gene (σ^32^) was constructed by PCR amplification from chromosomal DNA with primers JW2199 and JW2200 (Table 1). The PCR product was digested with *Nhe*I and *Sph*I and ligated into similarly digested pBAD18-Cm [34].

**Table 1:** List of oligonucleotides used in this work

**Name**	**Sequence**
JW071	ACCAAAGCCATGACAAAA
JW072	TGGCATAGCAAAGTGTGA
JW073	CGCAGCAATTTAATCCAT
JW074	CCTGCCAGCAGAGAGTAA
JW075	TGCGATGTGATATTGCTC
JW076	TAGGGCAAAAACGAATGA
JW125	AAGCGAAAATCGGCAATA
JW126	CATGGCCTGCAACATATC
JW389	CGCGAACATCTTTTAACC
JW390	CAACGCCATAGACGACAT
JW393	GTCAACGCTTTATGGACTG
JW394	ACGAAGAGAAATAAGTGGATGT
JW1312	TTCGCTGTTACCTCTGGAA
JW1313	TTGGCCGATGATGGTTAT
JW1610	ACGTTTCGCCCCTATTAC
JW1611	ACCCAGGTCGATACCAAT
JW1612	GGTAGGTCGCGTTCCTT
JW1613	GAGGCGTGAACATGAGAT
JW1614	GCGTCGATTTCACCATT
JW1615	GGTTTTCCGCTTTTTCAT
JW1616	ATACCCTTCTCGGCAGTT
JW1617	CGCCTAAACACAGGGATA
JW1622	ACACCTTCTGTCTTCAGCTC
JW1623	GCCAGGGGTAGAATATCTG
JW2199	CTAGGCTAGCGAGAGGATTTGAATGACTGAC
JW2200	CTAGGCATGCTTACGCTTCAATGGCAGCAC

### Cell growth

For heat shock ChIP experiments, 100 ml LB was inoculated with 1 ml of fresh overnight culture of MG1655 *rpoH*-NFLAG and cells were grown at 30°C at 225 rpm to an OD_600_ of 0.5-0.6. Cultures were split (40 ml each) for further incubation at either 30°C or 50°C for 10 minutes. For ChIP experiments involving overexpression of σ^32^, 40 ml LB supplemented with 30 μg/ml chloramphenicol was inoculated with 0.4 ml of a fresh overnight culture of MG1655 containing either pRB1 or pBAD18-Cm. Cells were grown at 37°C at 225 rpm to an OD_600_ of 0.7-0.8. Expression of *rpoH* from pRB1 was induced by the addition of 0.2% arabinose and further incubation at 37°C for 10 minutes. For ChIP of AraC, AMD187 was grown in LB at 37°C at 225 rpm to an OD_600_ of 0.6-0.8.

### Standard ChIP method

Cells were crosslinked by the addition of formaldehyde to a final concentration of 1% for 20 minutes. Formaldehyde was quenched with glycine (0.5 M final concentration) and cultures were pelleted by centrifugation. Pellets were washed twice with Tris-buffered saline (TBS; pH 7.5) and resuspended in 1 ml FA lysis buffer (50 mM Hepes-KOH, pH 7, 150 mM NaCl, 1 mM EDTA, 1% Triton X-100, 0.1% sodium deoxycholate, 0.1% SDS) supplemented with 4 mg/ml lysozyme. After a 30 minute incubation at 37°C, cells were chilled on ice and sonicated in 30 second on/off pulses for 30 minutes at 100% output using a BioRuptor Sonicator. Lysates were centrifuged for five minutes to pellet cell debris. The supernatant was transferred to a new tube, brought up to a final volume of approximately 2 ml, and frozen in 0.5 ml aliquots. 0.5 ml crosslinked, sonicated cell lysate was brought up to a final volume of 0.8 ml with FA lysis buffer. A 20 μl aliquot was removed for “input” DNA control sample. 25 μl of protein A-Sepharose beads (50% slurry in TBS) and either 1 μl anti-RNA polymerase beta subunit (Neoclone) or 2 μl anti-FLAG (M2 monoclonal; Sigma) was added to the lysate and incubated for 90 minutes at room temperature with gentle rotation. Beads were pelleted at 4000 rpm in a microcentrifuge for one minute and the supernatant was removed. Beads were resuspended in 700 μl FA lysis buffer, transferred to a Spin-X column (Corning) and washed for three minutes by rotation, centrifuged for 1 minute at 4,000 rpm in a microcentrifuge and the flow through discarded. The beads were washed in a similar fashion with 750 μl of each of the following: FA lysis buffer, FA lysis buffer 500 mM NaCl, ChIP wash buffer (10 mM Tris–HCl, pH 8.0, 250 mM LiCl, 1 mM EDTA, 0.5% Nonidet-P40, 0.5% sodium deoxycholate) and TE (10 mM Tris–HCl, pH 8.0, 1 mM EDTA). The Spin-X column was transferred to a fresh tube and the chromatin was eluted from the beads by addition of 100 μl ChIP elution buffer (50 mM Tris–HCl, pH 7.5, 10 mM EDTA, 1% SDS) and incubation at 65°C for 10 minutes. The eluate was collected by centrifugation for 1 min at 4,000 rpm in a microcentrifuge. Crosslinks were reversed for both the eluate and the input samples by incubation for 10 minutes at 100°C. DNA was purified using QIAgen PCR purification kit followed by elution in either 50 μl or 200 μl for the IP samples or 200 μl for the input samples. For AraC ChIP, Spin-X columns were omitted from this procedure when indicated in the figure. Note that data shown for AraC ChIP/qPCR with Spin-X columns will be presented elsewhere (Stringer, A.M., Currenti, S.A., Bonocora, R.P., Baranowski, C., Petrone, B.L., Singh, N., Palumbo, M.J., Reilly, A.E., Zhang, Z., Erill, I. and Wade, J.T.: Comprehensive genomic analysis of the *Escherichia coli* and *Salmonella enterica* AraC regulons; in preparation).

### Modified ChIP method described by Waldminghaus and Skarstad

ChIP was performed as above but with the following modifications: (i) 100 μl of post-immunoprecipitation supernatant was substituted for the “input” control DNA sample, (ii) no Spin-X columns were used, (iii) 1 μl RNase A (30 mg/ml) was added after elution and incubated for 2 hours at 42°C for both the input and immunoprecipitated DNA samples, (iv) 80 μl TE and 20 μl proteinase K (20 mg/ml) was added incubated for 2 hours at 42°C, (v) crosslinks were reversed by incubation overnight at 65°C, and (vi) DNA was purified by phenol/chloroform/isoamyl alcohol and chloroform/isoamyl alcohol extraction followed by ethanol precipitation. Note that aliquots from the same sonicated, crosslinked cell extract were used for both the standard and modified ChIP methods.

### qPCR

ChIP and input samples were analyzed by quantitative real time PCR using an ABI 7500 Fast real time PCR machine, as described previously [2]. Enrichment of ChIP samples was calculated relative to a control region within the transcriptionally silent *bglB* gene, and normalized to input DNA. Occupancy units represent background-subtractedfold-enrichment. Oligonucleotides used for real time PCR were JW125/JW126 (*bglB*), JW1610/JW1611 (*dnaK*), JW1612/JW1613 (*ygcI*), JW1614/JW1615 (*ybjX*), JW1616/JW1617 (*tdk*-*ychG*), JW1622/JW1623 (*b2084*), JW071/JW072 (*araB*), JW073/JW074 (*araE*), JW075/JW076 (*araF*), JW389/JW390 (*ytfQ*), JW1312/JW1313 (*dcp*), and JW393/JW394 (*ydeN*; Table 1). Note that primers for *ytfQ* produced primer dimers in qPCR for ChIP with an untagged strain (Additional file 1: Supplementary Data), so we were not able to assess enrichment of this region.

### Estimating null distributions for ChIP-chip datasets to calculate z-scores

Previous studies have analyzed ChIP-chip datasets based on the assumption that the distribution of actual ChIP-chip signals below the modal value closely matches the null distribution, and fits a normal distribution [20,21]. We determined the modal value for each ChIP-chip dataset and used all probes scoring below the mode to estimate the standard deviation of a null distribution, treating the mode as the mean. We used these mean and standard deviation estimates to calculate z-scores (i.e. number of standard deviations from the mean) for each probe.

### Assessment of the number of DSTs in intergenic regions

88% of the *E. coli* genome is genic. Of the 46 DSTs, 15 have peak probe coordinates that fall in intergenic regions. Note that some additional DSTs were classified as being “intergenic” due to the stringent criterion used in our earlier work [11] to account for incomplete probe coverage on the microarray. We used a Binomial Test to determine the probability that 15 of 46 DSTs would be located in intergenic regions if their genomic position was unbiased with respect to genes.

### Comparison of DST z-scores to those of all z-scores for waldminghaus and skarstad datasets

For each replicate dataset, we determined the z-score for each DST peak probe. We then determined z-scores for 1,000 randomly-selected probes from the complete dataset. We used a Mann–Whitney U Test to determine the probability that the z-scores for DST peak probes are not larger than those of randomly-selected probes.

## Abbreviations

ChIP: Chromatin Immunoprecipitation; TF: Transcription factor; RNAP: RNA Polymerase; IP: Immunoprecipitation; DSTs: Disputed σ^32^ Targets; TBS: Tris-buffered saline

## Competing interests

The authors declare that they have no competing interests.

## Authors’ contributions

RPB performed the experiments described in Figure 2 and the Additional file 1: Supplementary Data. DMF performed the experiment described in Figure 3. AMS performed the experiment described in Figure 4. JTW performed all other analyses. JTW wrote the paper with input from RPB and DMF. JTW conceived the study. All authors read and approved the final manuscript.

## Supplementary Material

Additional file 1 Control ChIP/qPCR data using an untagged strain. Click here for file
